# A Context Similarity-Based Analysis of Countries’ Technological Performance

**DOI:** 10.3390/e20110833

**Published:** 2018-10-31

**Authors:** Andrea Napoletano, Andrea Tacchella, Luciano Pietronero

**Affiliations:** 1 Institute for Complex Systems—CNR, Via dei Taurini 19, 00185 Rome, Italy; 2 International Finance Corporation—World Bank Group, Washington, DC 20433, USA; 3 Dipartimento di Fisica, Sapienza University of Rome, Piazzale Aldo Moro, 00185 Rome, Italy

**Keywords:** innovation, economic studies, machine learning

## Abstract

This work contributes to the literature in the field of innovation by proposing a quantitative approach for the prediction of the timing and location of patenting activity. In a recent work, it was shown that focusing on couples of technological codes allows for the formation of testable predictions of innovation events, defined as the first time two codes appear together in a patent. In particular, the construction of the vector space of codes and the introduction of the *context similarity* metric allows for a quantitative analysis of technological progress. Here, we move from that result and we show that, through *context similarity*, it is possible to assign to countries a score which measures the probability of being the first to patent a potential innovation. In other words, we show that we can not only estimate the likelihood that a potential innovation will be patented in the imminent future, but also forecast where it will be patented.

## 1. Introduction

The task of modeling and forecasting innovations has always been a fundamental problem in data science. What is an innovation? There are many definitions in the literature, and, consequently, many possible approaches to the task [1,2,3]. A well-known framework developed to model innovation is the Adjacent Possible by Stuart Kauffman. In this view, an innovation is a step further from the border of what is possible right now, which, in turn, opens up a new region of the Adjacent Possible. After each new discovery, the border of the Adjacent Possible moves further and new unthought possibilities emerge [4,5]. Kauffman’s model has been the basis for many works on innovation; however, the majority of them are not grounded on concrete data and focus more on describing rather than predicting [6,7,8,9,10,11,12,13,14,15,16].

In this paper, we make use of the expression *radical innovation* to clearly distinguish innovations from novelties. In particular, a radical innovation is a true never-seen-before event in the moment when it happens, a novelty is an innovation only in a restricted context, e.g., the discovery of a new song written 5 years ago is an innovation only for the person who hears it for the first time now, but not a true innovation because it already exists. In a recent work, we introduced an effective method to predict forthcoming radical innovations based on two simple assumptions (see [17,18]). First, to predict radical innovations we start by defining a framework, which, in our case, is the technological progress documented by the PATSTAT database [19] through technological codes [20]. Working on a concrete example allows us to pose and answer concrete questions, rather than study abstract models not grounded on real data. Second, the operative definition of innovation must be suitably chosen to overcome the obstacle that no algorithm can be trained to classify an event outside its training set. Since an innovation is by definition a never-seen-before event, when it happens, the system is not ready to classify it.

In our work, we have chosen to pursue the parallel with natural language, where neologisms usually first appear in the form of compounds. Indeed, a fundamental way in which humanity innovates is by combining pre-existing things to make them fulfill new functions [21,22]. For this reason, we define a radical innovation as the first combination of two technological codes together in a patent, rather than the ex novo introduction of a new technological code. We have shown that through natural language processing (NLP) techniques, such as the World2Vec algorithm [23,24], it is possible to build the vector space of technological codes. In such a space, a similarity metric between codes, which we refer to as *context similarity*, quantifies the contextual distance between codes, see Section Methods for a brief introduction on the subject. We have already shown in detail that looking at the dynamics in this space allows for the prediction of radical innovations with high accuracy (see [17,18]). In this work, we take this a step further and discuss how to predict where a radical innovation is more likely to be patented.

We assessed the technological production of a country by looking at the nationality of the owners of each patent. We made use of this information to estimate each country’s contribution to global patenting activity and to predict where it is more likely that a specific innovation event will occur. A localized economic analysis, which focuses primarily on individual nations, and a globalization approach, which considers external international relations, are already well developed in the literature [25,26,27]. Our work contributes to both directions, specifically in the field of technological innovation.

To conclude this introductory section, we give an overview of each country’s technological performance by looking at the percentage of radical innovations they patent, with respect to both the global patenting activity and the country’s patenting activity. In the next section, we move from a descriptive analysis to a predictive analysis and show how to forecast the radical innovations that each country will more likely patent.

PATSTAT provides the complete submission data of its patents, with information about the year, the month, and the day. However, to have statistically significant data, we grouped patents yearly. Coherent with this choice, when looking at the national patenting activity in different countries, we consider an innovation up-to-date if the country patents it in the same year in which it first appeared in the world. For this reason, an innovation can be up-to-date in different countries at the same time.

**Figure 1:**
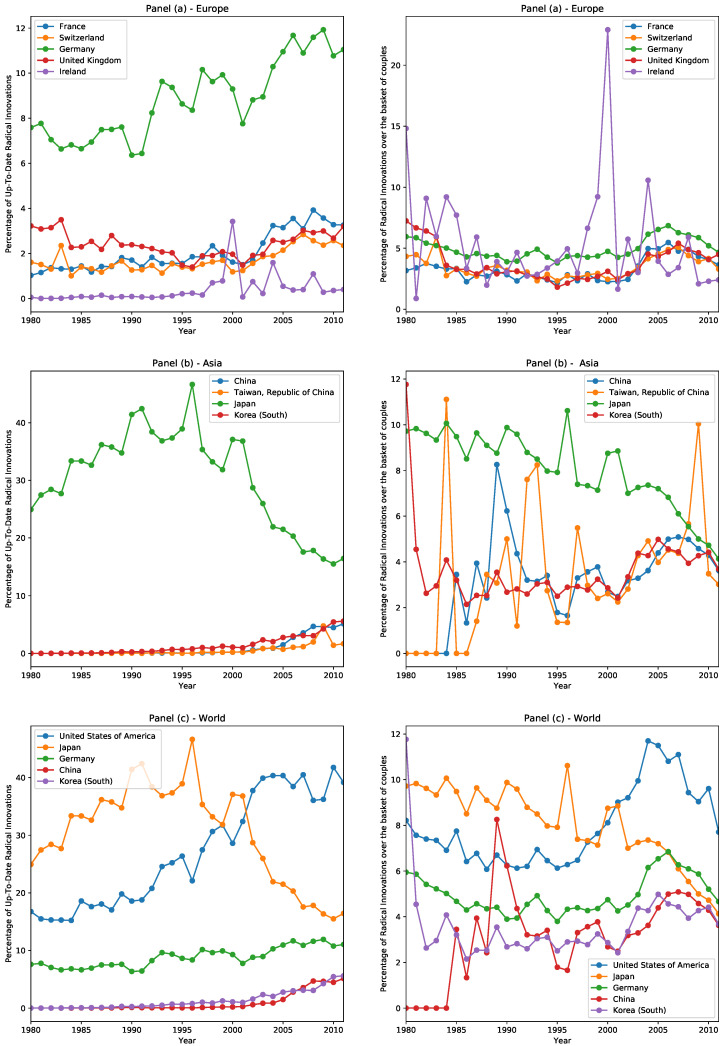
In each row, the left plot shows the percentage of radical innovation with respect to all radical innovation of that year, and the right plot shows the percentage of radical innovation with respect to the patenting activity of the country. Panel (**a**): Europe—In Europe, Germany occupies the first place, France, Switzerland, and the United Kingdom are in the middle, followed by Ireland at the bottom. Panel (**b**): Asia—In Asia, Japan is in a leading position but is experiencing a decline that started at the end of the last century; South Korea, Taiwan, and China are emerging and starting to catch up. Panel (**c**): World—Top innovating countries in the world are the United States of America, Japan, Germany, and, in recent years, China and South Korea.

We calculated, for each country, the year percentage of up-to-date innovations, i.e., the fraction of innovative patents with respect to all radical innovations patented in the world, and we ranked all countries according to such percentages. This measure can be used as a proxy to identify the most innovative countries. We show the results for the most innovative countries in Figure 1. Notice that the percentages shown do not sum to 100%. This is because, in this analysis, we are considering only the year in which a radical innovation was introduced and not the precise date. Therefore, if in country A and country B an innovation was patented with some months of delay but in the same year, both countries are considered up-to-date with respect to the state of the world. For this reason, different countries can share the same radical innovations and the sum over all countries can surpass 100%. In the left figure, we plot the aforesaid percentage, while, in the right figure, we plot the percentage of innovative couples of a country with respect to all its patented technological couples.

Just from this descriptive analysis, we gather some interesting facts. If we consider only the top 5 innovative countries each year, over the three decades from 1980 to 2011, we observe 11 countries: the United States and Canada in the American continent; Germany, the United Kingdom, France, Switzerland, and Ireland in Europe; China, Japan, South Korea, and Taiwan in Asia. In Figure 1, we focus on the European continent in panel (a), on the East in the panel (b), and only on the top countries in the panel (c).

- **Europe**: The European scene is dominated by Germany, followed by the United Kingdom, France, and Switzerland, which are close to one another in terms of shares of radical innovations. Ireland is in the bottom position among the top European countries. Recall that we assign the nationality of each patent, and, therefore, of each radical innovation, by looking at the nationality of the owner of the patent. It is possible that, due also to easier regulations and lower taxation, Switzerland and Ireland appear among the top innovative countries.
- **Asia**: Since the 1980s, Japan has been the leading country both in Asia and in the World. In this period of technological dominance, of all radical innovations realized every year in the world, Japan was up-to-date in at least 30% of them, with even a peak of almost 50%. Once it entered the new millennium, however, Japan experienced a decline of its fraction of innovative patents, settling roughly to 20%, while other countries emerged, such as China, Taiwan, and South Korea.
- **World**: The United States, which was in the second position below Japan until 2000, is since the world leader in technological progress. It is also noteworthy that Germany has always occupied the third position in the time range considered, showing a slightly increasing trend in up-to-date innovations.

*Context similarity* offers a way to pass from a merely descriptive analysis to a deeper understanding of technological progress. In what follows, we propose a way to identify which country is more likely to patent a radical innovation. We assign to each potential innovation a score depending on the specific country to measure its competitiveness. We show that such a score is a good predictor of the nationality of radical innovations, and then discuss how it is defined and calculated.

## 2. Results

The dynamic in the space of technologies provides enough information to successfully forecast radical innovations (see [17,18]). Here, we took this a step further and addressed the problem of predicting which country is more likely to introduce them. To achieve this goal, we assigned to each potential innovation a score depending on the specific country to measure its capability to patent the innovation. We refer to such a quantity as the Country Score (CS) and, to avoid ambiguity with *context similarity*, we always write the latter explicitly.

We focused on 10 sliding windows, covering a time range of almost 20 years, from the sliding window 1990–1999 to the sliding window 2000–2009. Each sliding window is composed of two non-overlapping subwindows: a 5-year-long training set used to calculate the *context similarity* and the Country Score of potential innovations, and the subsequent 5-year-long testing set, where we check if potential innovations are introduced. We restricted ourselves to potential innovations with high *context similarity*; in this way, on the one hand, we considered only couples which we already know are more likely to be patented, and on the other hand, we limited the computational time required to calculate the scores for a large number of couples. This restriction leaves us with around 3000 potential innovations per sliding window As we will explain in more details in the Methods section, 1500 of them are used as a validation set and the remaining 1500 are used as a testing set. Results discussed here were obtained using only the 1500 couples forming the testing set.

The Country Score plays two roles in our analysis. First, it allows us to perform an international analysis, ranking countries on the basis of their technological capabilities with respect to the basket of potential innovations selected. Second, it sheds light on each individual country, identifying, among potential innovations, those that are more easily achievable. Given the twofold nature of the Country Score, we performed two different tests to measure its effectiveness. We checked which potential innovation of a given training set becomes a radical innovation in the corresponding testing set and, then, we proceeded to analyze which country patents it in the same year. Indeed, we consider a country up-to-date with respect to a certain radical innovation if aforesaid country patents radical innovation in the same year when it gets patented for the first time in the world. We divided potential innovation into two classes: class 1 if the potential innovation becomes a radical innovation and the country patents it in the same year; class 0 otherwise (i.e., either if the potential innovation does not become a radical innovation, or if the country does not patent it in time.). In this way, for each country, we can associate to each potential innovation the Country Score, calculated in the training set, and its label, determined in the testing set. A sample of the data is shown in Table 1.

As stated before, the Country Score allows us to study the technological capabilities of a country, both compared to other countries and considered in isolation. Let us start with the first case: we want to test if the CS calculated in the training set is a good predictor of the labels assigned to potential innovations in the testing set. In other words, we want to test if the CS is able to predict where a certain potential innovation will be patented. Since most countries do not have significant patenting activity, we limited the analysis to only the most active ones described in the introduction of this work: United States, Canada, Germany, United Kingdom, France, Switzerland, Ireland, China, Japan, South Korea, and Taiwan. We compared the performance of the CS with a Baseline Model based on the diversification of the country’s patenting activity. In particular, let C be a country; we define the Baseline Model as the number of different couples patented by C in the training set.

BaselineModel=#C=NumberofdifferentcouplespatentedbyC.

**Figure 2:**
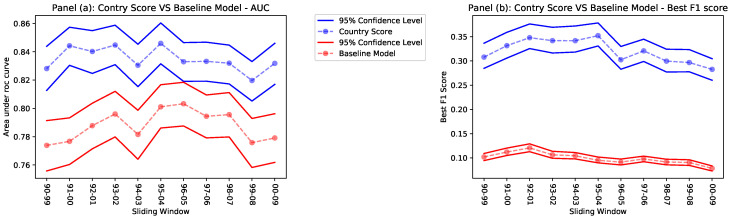
Performance evaluation of the CS compared to the Baseline Model, as measured by the area under the ROC curve (receiver operator characteristic curve) (AUC) with a confidence level of 95% in Panel (**a**) and, by the F1 Score in Panel (**b**).

Notice that, in this definition of the Baseline Model, the specific potential innovation does not appear: each country has the same score for all potential innovations, and, therefore, the Baseline Model, differently from the Country Score, can no be used within single countries. The reason behind this choice is straightforward: we want to test if *context similarity* can successfully identify a specific zone in the space of technologies where the patenting activity is more relevant for a given potential innovation, or if it is sufficient, to consider just patenting activity without any further distinction. In the first case, if a coherent patenting activity is important for introducing radical innovations, we expect the CS to perform better, while, in the second scenario, if all the relevant information is condensed in the diversification of patenting activity, we expect no significant advantage in using CS over the Baseline Model. Results of the comparison between the two classifiers are shown in Figure 2. We used the area under the ROC curve (AUC) and the best F1 score to evaluate the effectiveness of the CS and the Baseline Model as predictors of radical innovations. More details on their definition can be found in the Methods section and in the literature, e.g., [28,29,30]. Results are shown in Figure 2.

Both the AUC and the F1 score suggest that the CS outperforms the Baseline Model in correctly predicting the nationality of future radical innovations. In particular:

- The AUC, although showing that the CS performs better, is high for both predictors. We argue that having a diversified and florid patenting activity is a fundamental technological capability: countries with a greater patenting activity will more likely be protagonists of the majority of radical innovations.
- However, the best F1 score shows that the CS outperforms the Baseline Model since the latter loses precision and recall (see Methods section) because it fails to take into consideration the coherence of the patenting activity of a country and does not go into the details of each potential innovation.

The diversification of the technological production of countries, which we roughly estimate as the number of different technological couples patented, plays an important part: countries with well-developed patenting activity are more likely to patent innovations. However, diversification is not enough to understand which are the technological areas in which a country is more likely to progress and make innovations. As we argue in the Methods section, the Country Score makes a step further and analyzes each potential innovation, assessing the technological capabilities of a country relevant to that specific innovation, and in this way, it provides better forecasting performance. To conclude this section, we show how the CS can be effectively used to make predictions within single countries.

**Figure 3:**
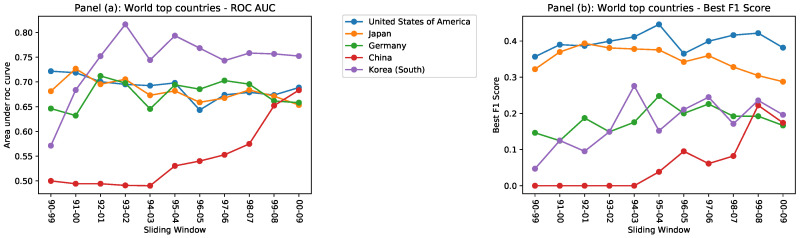
In panel (**a**), we show the ROC AUC for different countries as calculated across all sliding windows. In panel (**b**), we show the best F1 score for the same countries.

Figure 3 shows the predictive power of the Country Score evaluated respectively by the AUC and the best F1 score. We focus only on more technologically prominent countries: United States, Japan, Germany, and, in more recent years, China and South Korea. Both figures prove that the CS is a reliable instrument to forecast which radical innovations are more easily attainable for a country, given its current technological production.

## 3. Discussion

In the space of technologies, the position of each technological code is the result of the analysis conducted on all patents in the training set. Clusters of codes represent their real usage: codes more often used together are represented closer in the space of technologies, and *context similarity* is a measure of this contextual proximity. We have already shown how this metric can be effectively used to forecast radical innovations. This work moves forward and introduces the Country Score, which measures the capabilities of a country to patent certain innovations. The idea behind it is simple and straightforward: a radical innovation occupies a well-defined position in the space of technologies and it is surrounded in the immediate proximity by codes relevant to its technological area. To be able to patent it, a country must be active in that technological area, and the Country Score measures exactly this proficiency. We refer to the Methods section for more details.

**Figure 4:**
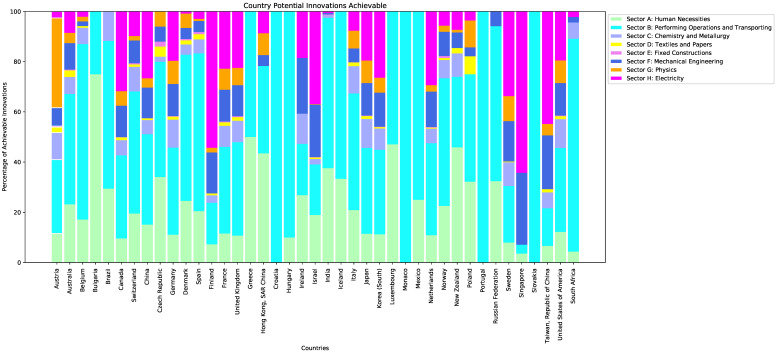
The plot shows the normalized percentage of the selected radical innovations that each country is able to patent according to the Country Score in the sliding window 2000–2009. Here, we are simply checking if the CS of a country is greater than 0 for the selected potential innovations, rather than considering the actual value of the CS.

In Figure 4, we show an example of an investigative application of the Country Score. For each selected potential innovation, we have calculated the CS for all countries and counted the percentage of potential innovations with a score greater than 0. We plotted this percentage calculated in the sliding window 2000–2009 while focusing on countries that have a nonzero CS. Each bar is normalized to 100 and highlights the technological sectors of the codes forming potential innovations available to each country, thus giving an idea about the technological areas in which the country is more proficient.

We believe that the work presented here is a natural application of *context similarity*, and the framework introduced can be used to conduct detailed studies of the technological capabilities of countries, also in relation to their economic and industrial performance. For example, the analysis shown in Figure 4 can be made more specific by selecting different radical innovations. Distinguishing radical innovations with respect to the technological sectors of their codes, we can rank countries’ competitiveness in different areas. Another possibility is to investigate deeper the different values of the AUC shown in Figure 3. It is reasonable to assume that countries in which the CS performs better have a more focused patenting activity, and it is worth investigating whether being more technologically coherent is associated with an advantage in terms of economic performance.

## 4. Materials and Methods

### 4.1. Context Similarity: A Brief Introduction

The PATSTAT database allows us to associate to each patent the list of technological codes it employs. We grouped patents into sets of 5 years and used them to train a neural network to learn contextual relations between technological codes. In particular, we considered the list of technologies employed by a patent as a context, and we randomly extracted one code and used it as input to teach the algorithm to predict the remaining elements of the *context*. At the end of the training, the internal vector representation of each code is representative of the contextual relations among them. We call such vectors embeddings. Let *C* be a technological code; we define $\vec{E}\left(C\right)$ as the embedding of the technological code *C*. On each training set, we trained 30 different copies of the same neural network, and we define the *context similarity* of codes $C_{1}$ and $C_{2}$ as the scalar product $S_{1,2}=\vec{E}\left(C_{1}\right)\cdot \vec{E}\left(C_{2}\right)$ averaged over 30 runs. For more details, see [17,18,23,24].

### 4.2. Country Score: Definition and Tuning

In this section, we discuss how to estimate the capability of a country to patent a potential innovation through *context similarity*. In principle, *context similarity* ranges between [−1,1], but we have empirically observed a distribution of values in the range [0,1], with 0 meaning that the two codes are as different as possible, and 1 meaning that they practically can be considered synonyms [17,18]. Recall that the higher the *context similarity*, the higher the probability of the potential innovation to be patented. Let us consider two technological codes *A* and *B*; we define the distance D(A,B) between codes A and B as:

D(A,B)=1−contextsimilarity(A,B)

where now D(A,B) close to 0 implies that the two codes are very similar, while D(A,B) close to 1 means that the two codes belong to unrelated technological areas.

Let us consider the space of technologies formed by the *N* embedded technological codes, and let A−B be a generic potential innovation under investigation. The position of the other embedded codes in such a space, or more precisely, the mutual distances between them and the innovation A−B, reflects how relevant they are in creating possible bridges between the unconnected couple A−B. The closer a technological code is to such a couple, the more influence it has on it, because their contexts are more semantically related.

**Figure 5:**
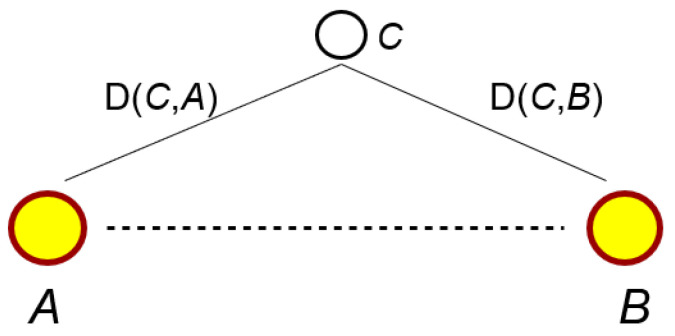
Paths for potential innovations. Code *C* is close to both code *A* and code *B*; therefore, a country that already has patents linking *A* and *C* and *B* and *C* is more likely to patent the potential innovation *A B* than a country that does not.

In Figure 5, for example, we show a specific code *C* which is close both to code *A* and code *B*: if a country possesses patents that link code *A* with code *C*, and code *B* with code *C*, it is likely that it has the capabilities to patent the potential innovation A−B.

Our assumption is that, in order to patent a potential innovation, a country must be competitive in the technological sectors involved. There is no established procedure to measure such a degree of competitiveness; therefore, we propose our own method. Let $C_{1}$ and $C_{2}$ be two technological codes; for $C_{1}\neq C_{2}$, we define:

$$\begin{array}{c} E^{C}\left(C_{1},C_{2}\right)=1\;\mathrm{If}\;\mathrm{country}\;C\;\mathrm{has}\;\mathrm{at}\;\mathrm{least}\;a\;\mathrm{patent}\;\mathrm{linking}\;C_{1}\;\mathrm{and}\;C_{2}\\ E^{C}\left(C_{1},C_{2}\right)=0\;\mathrm{Otherwise} \end{array}$$

while, if $C_{1}=C_{2}$, $E^{C}\left(C_{1},C_{2}\right)=0$. Given a potential innovation A−B, let us call $C_{i}$ a generic technological code among the *N* embedded ones; for each country C, we check if there are patents employing the couple $A-C_{i}$ and patents employing the couple $B-C_{i}$. In other words, we build the *N*-dimensional vector $\vec{B^{C}}\left(A,B\right)$ whose components $B_{i}^{C}\left(A,B\right)$ are defined as:

$$B_{i}^{C}\left(A,B\right)=E^{C}\left(A,C_{i}\right)\times E^{C}\left(B,C_{i}\right).$$

By definition, $B_{i}^{C}=1$ if country C has at least one patent linking *A* with $C_{i}$ and one patent linking *B* with $C_{i}$, and it is 0 otherwise. Then, we define the vector of weights $\vec{W}\left(AB\right)$ as a Gaussian kernel through its components:

$$W_{i}\left(A,B\right)=e^{-\frac{\left(D{\left(A,C_{i}\right)}^{2}+D{\left(B,C_{i}\right)}^{2}\right)}{2\sigma_{AB}^{2}}}$$

where $\sigma_{AB}$ is a free parameter that dictates the scale of the problem. In particular, we set $\sigma_{AB}$ proportional to D(A,B):

$$\sigma_{AB}=\alpha \times D\left(A,B\right)$$

To lighten the notation, we suppress the dependence from A−B and leave it implicit. For each potential innovation, we assign to a country C the score S(C), calculated as:

$$S\left(C\right)=\frac{\vec{B^{C}}\cdot \vec{W}}{\sum_{i}^{N-2}W_{i}}.$$

**Figure 6:**
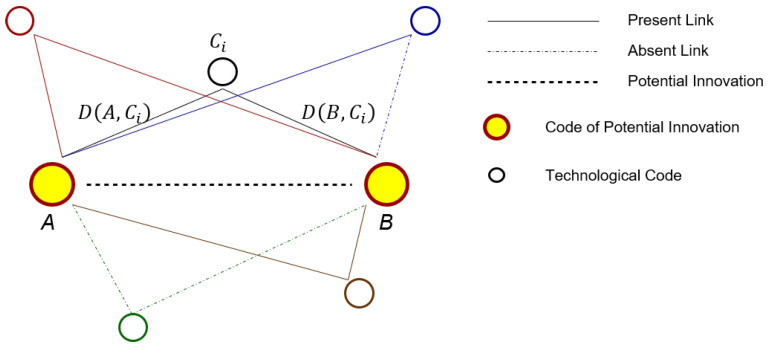
Country Score. The diagram above illustrates the procedure we use to assign a score to each country for each potential innovation. Such score measures how active is a country in those technological sectors that influence the closeness of the contexts of the potential innovation. The score is defined as the weighted average of all indirect 3-codes-long paths joining the potential innovation.

This score has an immediate geometric interpretation, and Figure 6 helps to understand it. For every code $C_{i}$, we consider the path $A-C_{i}-B$ that links *A* to *B*: in a given country, such a path exists if and only if the country has both parts of the path $A-C_{i}$ and $B-C_{i}$, i.e., if it has at least a patent with the couple $A-C_{i}$ and a patent with the couple $B-C_{i}$. The score S(C) is the average weight of closed paths, where each of them is weighted by its length through the weights vector $\vec{W}$. The more three-codes-long paths from *A* to *B* a country possesses, the more likely it will patent the direct link A−B.

**Figure 7:**
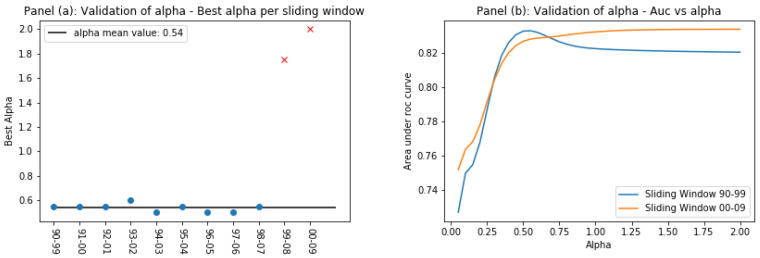
Tuning of α. Panel (**a**) shows the best α in each sliding window, defined as the one yielding the highest area under the ROC curve. Panel (**b**) shows the curve AUC vs. α in two sliding window: the first is 1990–1999 and the last is 2000–2009. In the last window, due to a blurring of technological context, it is not possible to identify the best-performing α.

The parameter α defines the scale of the problem, and we propose to tune it with an out-of-sample calibration. As we state in the introduction of this work, we have an average of 3000 potential innovations per sliding window. With a random extraction, we randomly selected half of them to test the CS against the Baseline Model and used the remaining ones as a validation set to tune the parameter α. Since the choice of α impacts the prediction power of the CS, which can be measured through the area under the ROC curve, we chose the best α in the validation set as the one that provides the highest AUC, and then used it in the testing set. In Figure 7, we show the result of tuning α in each sliding window. In panel (a), we show the evolution of the best α, while, in panel (b), we show two examples of the AUC as a function of α.

Setting aside the outliers, panel (a) of Figure 7 clearly shows that the best α can be consistently approximated with 0.55 throughout the entire time period considered. The last two sliding windows, on the contrary, do not show the same behavior. Panel (b) shows that this is due to a drastic change in the dependency of the AUC as a function of α. Where in the previous sliding windows there was a clear peak to identify the best performing α, in the last two, the AUC increases as α grows. We believe that we can trace this phenomenon back to a change in the nature of the data that we discussed in [17,18] and to which we refer for a detailed treatment, while here we provide a brief description of what we believe happens.

From 2004 to 2006, there was an explosion of patented radical innovations per year in the world, due to an increase in the number of technological codes per patent that stopped after a major reclassification of technological codes in 2006. This has had a tangible effect on the network of technologies, which, in these years, is less clusterized and more similar to a fully connected network. For this reason, the technological contexts captured by *context similarity*, and on which the Country Score is based, are more blurred. We argue that this is the reason behind the change in shape of the curve shown in panel (b) of Figure 7: since technological contexts are less well defined, technological codes further away from the potential innovation have a greater impact on it. Given the impossibility to tune α in the last two sliding windows with the method employed for the previous ones, we have decided to use the value of 0.55 in all the time intervals considered, ignoring the outliers. Figure 2 shows that, despite this choice, the Country Score still outperforms the Baseline Model.

### 4.3. ROC and F1 Score

In this section, we briefly describe how to calculate the ROC curve and the best F1 score employed to evaluate the performance of the Country Score and the Baseline Model.

The ROC curve is drawn based on the four possible outcomes in a binary classification problem. First, potential innovations are sorted according to their Country Score; then, a moving threshold in the ranking is used to classify all potential innovations with a rank higher than the threshold as class 1, and the remaining innovations as class 0.

1. If the outcome from a prediction is class 1, and the actual value is also class 1, then it is called a true positive (TP).
2. If the outcome from a prediction is class 1, but the actual value is class 0, then it is said to be a false positive (FP).
3. If the outcome from a prediction is class 0, and the actual value is also class 0, then it is called a true negative (TN).
4. If the outcome from a prediction is class 0, but the actual value is class 1, then it is said to be a false negative (FN).

Once all predictions have been checked and classified into one of these four classes, the True Positive Rate (TPR) and False Positive Rate (FPR) are evaluated as follows:

$$TPR=\frac{TP}{TP+FN}\;FPR=\frac{FP}{FP+TN}.$$

The couple (TPR, FPR) represents a point in the False Positive–True Positive plane. The ROC curve is obtained by moving the threshold starting from the last element in the ranking up to the first. The area under this curve measures the effectiveness of the Country Score as a classifier: an area of 0.5 corresponds to the performance of a random classifier, while an area of 1 corresponds to a perfect classifier.

The measurement of the area under the ROC curve can be integrated with the calculation of the related confidence interval. In particular, we calculate the confidence interval corresponding to a confidence level of 95%. We evaluate this interval through a bootstrap of the ROC curve characterized by 1000 extractions, replacing the number of “CS-label” couples equal to the length of the original vector [31].

The F1 score is defined as the harmonic average of precision and recall of the classifier. Let *p* be the precision of the classifier, and let *r* be its recall; then:

$$p=\frac{TP}{TP+FP}\;r=\frac{TP}{TP+FN}=TPR.$$

The F1 can be calculated as:

$$F1=\frac{2}{\frac{1}{p}+\frac{1}{r}}=2\frac{pr}{p+r}$$

The calculation of the F1 score requires actual predictions of the labels that potential innovations will assume in the testing set. To transform the CS and the Baseline Model into predictions, we introduced a threshold for both of them and assigned label 1 to all potential innovations with a score higher than the threshold, and label 0 otherwise. Moving the threshold from the lowest score to the highest, we calculated different F1 scores and considered the highest one to evaluate the performance of the classifiers. Finally, with the same procedure employed for the ROC curve, we calculated the confidence interval of 95% associated with the best F1 score.

## Figures and Tables

**Table 1:** Country Vector. We assigned to each country its Country Score (CS) and a label equal to either 1, if the country patents the radical innovation, or 0 otherwise.

Potential Innovation	CS	Label
Code A–Code B	Score(A B)	1
Code A–Code D	Score(A D)	0
Code A–Code F	Score(A F)	0
Code B–Code C	Score(B C)	1
Code B–Code D	Score(B D)	1
Code C–Code D	Score(C D)	0

## Abbreviations

The following abbreviations are used in this manuscript:

ROC	Receiver Operator Characteristic curve
AUC	Area under ROC Curve
CS	Country Score
NLP	Natural Language Processing
TP	True Positive
TN	True Negative
FP	False Positive
FN	False Negative
TPR	True Positive Rate
FPR	False Positive Rate
p	Precision
r	Recall
